# Does Health Literacy Mediate Sociodemographic and Economic Inequalities in Fruit and Vegetable Intake? An Analysis of Slovenian National HLS19 Survey Data

**DOI:** 10.3390/foods14030378

**Published:** 2025-01-24

**Authors:** Andrej Kirbiš, Stefani Branilović, Maruša Lubej

**Affiliations:** Department of Sociology, Faculty of Arts, University of Maribor, 2000 Maribor, Slovenia; stefani.branilovic@student.um.si (S.B.); marusa.lubej2@um.si (M.L.)

**Keywords:** health literacy, fruit and vegetable intake, mediation analyses, demographic and socioeconomic inequalities

## Abstract

Health literacy plays an important role in promoting healthier behaviors. However, less is known about its impact on dietary habits, such as fruit and vegetable (FV) intake. This study examines the mediating role of health literacy in the relationship between FV intake and demographic and socioeconomic factors among Slovenian adults. We used Slovenian national data from the 19-country Health Literacy Survey 2019–2021 (HLS19) (*n* = 3360). Results indicate that health literacy plays a complex role in mediating the relationship between demographic and socioeconomic factors and FV intake. Women and financially stable individuals have higher levels of health literacy, which positively predicts higher FV intake. While older individuals demonstrated greater FV intake, they reported lower health literacy, suggesting other mechanisms may drive their dietary behavior. Additionally, while higher education was associated with improved health literacy, its direct relationship with FV intake was negative, though health literacy partially mitigated this effect. These results underscore the importance of health literacy as a mediating factor in improving dietary behaviors and highlight the need for targeted interventions and policies to enhance nutritional education, particularly for marginalized groups.

## 1. Introduction

Diet plays a critical role in health and longevity. Evidence consistently shows that food intake is the leading cause of premature mortality and non-communicable diseases, including cardiovascular disease, cancer, and diabetes [1,2,3,4]. Recognizing this, the World Health Organization has emphasized diet as a key determinant of disease risk and has developed strategies to prevent and control diet-related health issues by addressing unhealthy eating patterns [4,5]. Despite this, significant inequalities in dietary patterns persist, influenced by demographic, economic, and other factors. Understanding these disparities is crucial for designing effective interventions to improve diet and reduce health inequities.

Previous studies have highlighted several demographic and economic factors of health, such as gender, age, education, and economic status. Gender differences, in particular, have been shown to influence health outcomes, with women generally experiencing lower rates of disease [6,7] and having longer life expectancy compared to men [8]. These differences can be partly attributed to women’s lower engagement in risky behaviors and greater adoption of healthier lifestyles. For instance, women are more likely to follow dietary recommendations, have greater health-related knowledge, and rely on diverse information sources for health-related decisions [9,10]. Women also report higher consumption of fruits and vegetables compared to men [11], a gender gap that has increased over the past several decades [12]. In contrast, men’s lower fruit and vegetable intake has been linked to behaviors that reflect traditional masculinity norms, such as engaging in riskier behaviors to assert dominance and reinforce their identity as men [13]. These gendered differences underscore the importance of addressing social and cultural factors that shape dietary patterns and contribute to health inequalities.

Compared to younger adults, older individuals tend to experience higher mortality rates and poorer overall health, largely due to the biological processes of aging. However, healthier dietary choices among the elderly may mitigate some of these age-related health challenges. For example, a systematic review by Milte and McNaughton [14] found that diets rich in fruits, vegetables, and whole grains are associated with better cognitive function, mental health, and improved quality of life among older adults. Moreover, studies have shown that fruit and vegetable consumption tends to increase with age [15,16], potentially reflecting a greater awareness of health benefits or increased adherence to dietary guidelines. In contrast, younger populations are more likely to exhibit unhealthy eating behaviors, such as consuming high-fat snacks, eating nighttime meals, and overeating [17]. The Determinants of Diet and Physical Activity (DEDIPAC) project found that 44% of female and 36% of male European adolescents skip breakfast, and among these “breakfast skippers,” fruit intake was significantly lower [18].

Education is another key determinant of health outcomes. Higher levels of education are consistently associated with greater life expectancy and lower rates of both all-cause and specific mortality [6,15,19]. This relationship is mediated by several mechanisms, including the adoption of values that prioritize preventive health behaviors [15,20]. Education plays a critical role in improving diet quality by enhancing individuals’ ability to access, understand, and act on health-related information. For instance, studies indicate that higher education levels are linked to higher fruit and vegetable consumption among European populations [11,21]. Lower socioeconomic position, particularly low education, is associated with inadequate intakes of nutrients essential for a healthy diet, including higher cholesterol intake and lower intakes of fiber, vitamin C, and beta-carotene [22]. However, in countries where fruit and vegetable availability and intake are already high, this association may be weaker or even reversed [23]. These findings suggest that education not only influences individual dietary choices but also interacts with broader societal and environmental factors, underscoring its complex role in shaping health outcomes.

Living in poorer economic conditions significantly worsens health outcomes, increasing the likelihood of cardiovascular disease, obesity, and other chronic conditions [19,24,25]. Economically disadvantaged groups, such as unskilled workers, the unemployed, and individuals receiving disability pensions, also experience significantly higher mortality rates [26]. One key explanatory mechanism for these disparities is dietary behavior, as individuals in higher economic groups are more likely to adhere to nutritional guidelines [15,27,28]. These groups report greater adherence to healthy dietary patterns, including higher consumption of fruit and vegetables [16,29,30]. In contrast, economically disadvantaged individuals consumed less magnesium, potassium, folate, and vitamin C [22].

Finally, health literacy has increasingly been recognized as a critical determinant of health and mortality. Health literacy encompasses a set of skills that enable individuals to effectively navigate the healthcare environment, including the ability to read, understand, and interpret health information [31,32]. Research demonstrates that higher health literacy is associated with better health outcomes [33], lower mortality rates [34], and increased life expectancy [35]. Additionally, studies have linked health literacy to healthier lifestyle behaviors. For instance, individuals with low health literacy are more likely to smoke, engage in insufficient physical activity, be overweight, and report poorer physical and mental health [2]. These improved outcomes among individuals with high health literacy may be partially attributed to enhanced health-related knowledge, greater self-efficacy, and reduced stigma surrounding health conditions, all of which empower individuals to make healthier choices [36].

Health literacy (HL) has also been examined as a potential factor influencing dietary choices and food intake [3,37,38]. For example, Cha et al. [39] found that HL predicts the use of food labels, which in turn improves dietary quality. Individuals with higher HL levels consume fewer sugar-sweetened beverages [40] and more frequently include fruits and vegetables in their diets [3,41,42,43]. Reflecting the critical importance of HL, the American Dietetic Association has identified it as a top priority in health and labeled it a “mega issue” in the field of dietetics [40]. Given HL’s pivotal role in shaping health outcomes and fruit and vegetable (FV) intake, as well as demographic and economic disparities in HL levels [28,44,45], it is essential to investigate whether inequalities in FV intake may be explained by group differences in HL.

Despite the growing evidence of HL’s importance for dietary patterns, several critical gaps remain in the literature. First, most studies on the relationship between HL and dietary behaviors, including FV intake, have focused on students and young people [38,43,46,47]. However, the role of HL in the diets of the general adult population has rarely been systematically explored [3]. Second, much of the research has been conducted outside Europe, primarily in non-European countries [3,38,40,46]. The limited number of studies conducted in Europe [6,41,48,49] restricts the generalizability of findings across European populations. Third, the present study uses the HLS19-Q47, an adapted version of HLS-EU-Q47 questionnaire, both comprehensive 47-item measures of HL that have been validated in adult populations across Europe [50,51], including Slovenia [52,53,54], and globally [55,56,57]. This comprehensive measurement responds to earlier calls by Carbone and Zoellner [58] for adopting broader conceptualizations of health literacy, moving beyond the narrower frameworks that have often been employed in previous studies. Finally, and most critically, previous research has primarily treated HL as a determinant of food intake [3,43] but has not explored whether HL explains demographic and economic inequalities in dietary patterns, such as FV intake, using causal models like mediation analysis. Scholars have identified examining HL as a mediator of dietary outcomes as a critical gap in the field [58].

This study aimed to investigate the following three key questions:

1. The direct link between health literacy and fruit and vegetable (FV) intake: Does higher health literacy lead to more frequent FV consumption?
2. Demographic and economic patterns of FV intake in Slovenia: Do inequalities in FV intake among Slovenian adults mirror those observed in other Western countries based on demographic and socioeconomic factors?
3. The mediating role of health literacy: Does health literacy explain the differences in FV intake between demographic and socioeconomic groups?

This study tested the following hypotheses:

**H1.**  *Female gender is associated with higher health literacy, which mediates the relationship between gender and higher levels of fruit and vegetable (FV) intake*.

**H2.**  *Older age is associated with higher health literacy, which mediates the relationship between age and higher levels of FV intake*.

**H3.**  *Higher education levels are associated with higher health literacy, which mediates the relationship between education and higher levels of FV intake*.

**H4.**  *Higher economic status is associated with higher health literacy, which mediates the relationship between economic status and higher levels of FV intake*.

## 2. Methods

### 2.1. Sample

We used Slovenian national data from the 19-country Health Literacy Survey 2019 (HLS19). Data were gathered between March 2020 and August 2020 using a probability sampling method with two-stage sampling from the Central Population Register. Respondents were asked to complete an online questionnaire, while some provided paper responses due to lack of online access. The sample included residents of Slovenia aged 18 or older and was weighted by gender, age, statistical region, and education. Of the 6000 individuals invited to participate, the final sample included 5585 individuals, yielding a 60% response rate, with 3360 completed questionnaires (for more details on sampling, see [52]).

### 2.2. Measurement

#### 2.2.1. Demographic and Socioeconomic Factors

For our independent variables, we used demographic and socioeconomic measures. We included gender (1 = male, 2 = female), age (recorded in years), education level (ranging from 1 = no formal education to 11 = master’s/doctorate), and economic status, measured with the question on economic deprivation: “How easy or difficult is it for you financially to pay bills for living expenses every month?” (1 = very easy; 4 = very difficult).

#### 2.2.2. Fruit and Vegetable Intake

The dependent variable in our study was fruit and vegetable intake, measured with the following question: “How many days in a typical week do you eat fruit, vegetables, or salad (excluding potatoes, freshly pressed fruit and vegetable juices, and juices from concentrate)?” (0 = not at all; 8 = 7 days).

#### 2.2.3. Health Literacy

The mediator in our study was health literacy, measured with HLS19-Q47. This questionnaire included three factors of health literacy: health care/treatment, disease prevention, and health promotion. Each factor was further divided into four dimensions: accessing health information, understanding health information, appraising health information, and using health information [59,60]. Items were measured with a four-point scale (1 = very difficult; 4 = very easy). Respondents’ health literacy was calculated by summing all responses to the statements, resulting in a score from 0 to 100. Higher scores indicated greater health literacy [52].

### 2.3. Statistical Analysis

**Figure 1:**
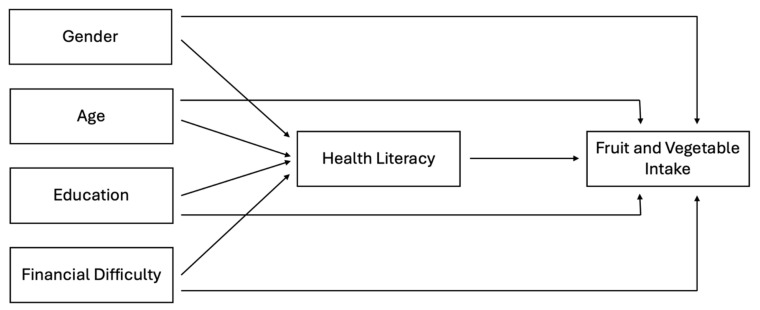
Conceptual model.

For our analysis, we used SPSS software (version 29.0.0.0 (241)) and R (version 2024.09.0+375). First, we used descriptive statistics to examine the characteristics of the sample included in our study (M, SD), and we continued with bivariate analysis, following Pallant [61]. For the main analysis, we used separate mediation models to research how health literacy explains the relationship between gender, age, education, and ability to pay bills as independent variables and levels of fruit and vegetable intake as a dependent variable (see Figure 1).

To ensure the validity of the mediation analysis, we checked various regression assumptions and two were not met. The Shapiro–Wilk test indicated deviations from normality for both the mediator and outcome models across all predictors (W ranging from 0.97 to 0.73, *p* < 0.01). Additionally, homoscedasticity tests (Breusch–Pagan) revealed heteroscedasticity for all predictors (*p* < 0.01). Based on these findings, the robust maximum likelihood estimator (MLR) was used in all mediation analyses to address deviations from normality and heteroscedasticity [62], ensuring robust and reliable parameter estimates. Additionally, we performed a mediation analysis with all predictors in one model to reduce the risk of inflated Type I errors from multiple comparisons [63,64]. The single model also allowed us to examine the combined and adjusted effects of all predictors simultaneously. The results of the single mediation model were similar to the separate mediation analyses and are available in Supplementary Materials. Unstandardized estimates are reported, and the significance level was set at 0.05.

## 3. Results

### 3.1. Sample Demographics and Socioeconomic Characteristics

Table 1 summarizes the sample demographics and socioeconomic characteristics. In the sample, there were 46.3% males and 53.7% females. In terms of education, most of the respondents had completed secondary technical education (25.3%), followed by lower or secondary vocational education (17.8%) and primary education (12.8%). Most of the respondents in the sample estimate that they can easily pay for monthly living expenses (52.5%), while a smaller percentage (34.8%) reported difficulty paying their living expenses.

### 3.2. Relationships Between Demographic and Socioeconomic Factors, Health Literacy, and Fruit and Vegetable Intake

Next, we examined bivariate correlations between key study constructs, including sociodemographic variables and financial strain. Correlation coefficients indicate significant relationships among the variables analyzed. The results are presented in Table 2.

Health literacy is higher among females (r_pb_ = 0.05, *p* < 0.01), younger individuals (r = −0.23, *p* < 0.01), those with higher education (ρ = 0.29, *p* < 0.01), and those with fewer financial difficulties (ρ = −0.27, *p* < 0.01). Average weekly fruit and vegetable intake is higher among females (r_pb_ = 0.14, *p* < 0.01), those with fewer economic difficulties (ρ = −0.08, *p* < 0.01), and individuals with higher health literacy scores (ρ = 0.08, *p* < 0.01). Fruit and vegetable intake is higher among older individuals (ρ = 0.19, *p* < 0.01) and those with lower education (ρ = −0.05, *p* < 0.01), with both relationships being weak but significant. Additionally, we performed separate Spearman’s correlation analyses for male and female participants, with the corresponding tables presented in Table S2. The results for both subgroups were consistent with those obtained for the overall sample.

### 3.3. Mediating Effects of Health Literacy on Demographic and Socioeconomic Predictors of Fruit and Vegetable Intake

We assessed the role of health literacy in explaining relationships between demographic and socioeconomic predictors and fruit and vegetable intake using mediation analyses. Table 3 summarizes the results, including direct effects, indirect effects via health literacy, and total effects. Below, we describe the findings for each predictor.

Gender significantly predicted health literacy, with females having higher health literacy scores than males (β = 1.137, 95% CI [0.176, 2.098], *p* < 0.05). Health literacy, in turn, positively predicted fruit and vegetable intake (β = 0.011, 95% CI [0.007, 0.015], *p* < 0.01). Gender directly predicted fruit and vegetable intake positively (β = 0.480, 95% CI [0.362, 0.598], *p* < 0.01), and the indirect effect through health literacy was also positive (β = 0.012, 95% CI [0.001, 0.024], *p* < 0.05). The total effect of gender on fruit and vegetable intake (β = 0.492, 95% CI [0.374, 0.611], *p* < 0.01) suggests that both direct and indirect pathways contribute to the higher intake in females. These findings confirm Hypothesis 1, demonstrating that females exhibit higher health literacy than males, which partially mediates their higher fruit and vegetable intake.

Age negatively predicted health literacy (β = −0.206, 95% CI [−0.233, −0.179], *p* < 0.01), with older adults reporting lower health literacy levels. However, health literacy positively affected fruit and vegetable intake (β = 0.018, 95% CI [0.013, 0.022], *p* < 0.01). The direct effect of age on fruit and vegetable intake was positive (β = 0.019, 95% CI [0.016, 0.022], *p* < 0.01), while the indirect effect through health literacy was small, negative, and significant (β = −0.004, 95% CI [−0.005, −0.003], *p* < 0.01). The total effect (β = 0.015, 95% CI [0.012, 0.018], *p* < 0.01) indicates that older individuals consume more fruits and vegetables overall, despite their lower health literacy. We do not find support for Hypothesis 2, as the results demonstrate that age is negatively associated with health literacy.

Education positively predicted health literacy (β = 1.929, 95% CI [1.689, 2.169], *p* < 0.01), with higher education levels associated with better health literacy. Health literacy, in turn, positively predicted higher fruit and vegetable intake (β = 0.013, 95% CI [0.008, 0.017], *p* < 0.01). However, the direct effect of education on fruit and vegetable intake was significantly negative (β = −0.030, 95% CI [−0.058, −0.001], *p* < 0.05), suggesting that higher education is associated with lower intake. The indirect effect of education on intake via health literacy was positive and significant (β = 0.025, 95% CI [0.016, 0.034], *p* < 0.01), but the total effect of education on fruit and vegetable intake was not significant (β = −0.005, 95% CI [−0.033, 0.023], *p* = 0.725). These findings provide partial support for Hypothesis 3, showing that education was linked to intake indirectly through health literacy but lacks a significant total effect.

Lastly, financial difficulty was a strong negative predictor of health literacy (β = −5.740, 95% CI [−6.473, −5.007], *p* < 0.01), with individuals facing more economic hardship reporting lower health literacy. Health literacy was positively associated with fruit and vegetable intake (β = 0.009, 95% CI [0.004, 0.013], *p* < 0.01). Financial difficulty also had a direct negative effect on fruit and vegetable intake (β = −0.166, 95% CI [−0.256, −0.076], *p* < 0.01) and a negative indirect effect through health literacy (β = −0.049, 95% CI [−0.075, −0.023], *p* < 0.01). The total effect (β = −0.215, 95% CI [−0.303, −0.127], *p* < 0.01) additionally showed the disadvantage of financial difficulty on dietary behavior. The hypothesis is supported, demonstrating that economic status is positively associated with health literacy, which mediates the relationship between economic status and fruit and vegetable intake.

## 4. Discussion

In the present study, we analyzed the relationship between socioeconomic and demographic differences and fruit and vegetable intake, mediated by health literacy. Previous studies on this topic emphasize that higher health literacy levels play a significant role in dietary intake, positively impacting fruit and vegetable consumption [3,42,43]. Moreover, studies examining the role of demographic and socioeconomic differences in health literacy and fruit and vegetable intake show that females, highly educated older adults, and economically stable individuals have higher levels of both fruit and vegetable consumption and health literacy [6,24,38,65]. However, few studies have empirically investigated the mediating role of health literacy between demographic and socioeconomic factors and fruit and vegetable consumption. Therefore, the aim of our study was to investigate how health literacy mediates the relationship between socioeconomic and demographic factors and fruit and vegetable intake.

In general, we found that higher health literacy levels are associated with greater fruit and vegetable consumption. Higher levels of fruit and vegetable consumption and health literacy are also found among females and economically stable individuals, while the results for age and education suggest a more complex relationship. Mediation analyses partly confirmed that health literacy mediates the relationship between demographic and socioeconomic status variables and fruit and vegetable intake levels. Female gender positively predicts higher health literacy levels, which in turn leads to higher levels of fruit and vegetable intake; the direct, indirect, and total effects all showed significant, positive relationships. This aligns with previous studies (e.g., [7,21,65]), which suggest that socially constructed gender roles—associating health risk behaviors with maleness and health-protective behaviors with femaleness—contribute to gender differences in health behaviors. Because of the acceptance of these roles and norms, men are particularly at risk of premature death from diseases influenced by health behaviors [8,13]. Women therefore show more positive attitudes toward healthy eating habits and higher levels of health literacy [45].

Secondly, we found that financial instability has a significant negative direct effect on fruit and vegetable consumption and a significant negative indirect effect through health literacy. The total effect was significant and negative. Our results are consistent with previous studies showing that individuals with financial difficulties have lower health literacy and lower levels of fruit and vegetable consumption, as they often do not follow daily nutrient recommendations or cannot afford healthier, higher-quality products (e.g., [20,28,66,67]).

Thirdly, the results of the mediation analysis with age as the main independent variable did not confirm findings from previous studies, as our results showed that higher health literacy levels are more common among younger people. In contrast, the direct effect of age on fruit and vegetable consumption was positive, while the indirect effect of age on fruit and vegetable consumption via health literacy was negative and significant. However, the total effect was still significant and positive, indicating that the positive direct effect of age on fruit and vegetable consumption outweighed the negative, indirect effect mediated by health literacy. This means that younger people have higher levels of health literacy, while older individuals have higher levels of fruit and vegetable consumption, regardless of their health literacy levels. The results are therefore partly inconsistent with previous studies. It seems that older people, in contrast to younger people, do have healthier dietary habits (e.g., [15,68]), but these habits are not connected to higher levels of health literacy.

One explanation may lie in cultural background and social influences, which can play a significant role in shaping dietary behaviors. For example, older adults may follow traditional diets rich in fruits and vegetables due to cultural practices and social expectations [69], rather than because of their health literacy. Social factors also play a role, as higher fruit and vegetable consumption is associated with greater social interaction and companionship, reflecting the central role of food in gatherings [70,71]. Furthermore, many older Slovenians reside in rural areas [72], where fruits and vegetables are more accessible and affordable, for example, through personal gardens or local markets [73]. Moreover, additional factors, such as travel time to grocery stores, price, and taste can also influence eating patterns among older adults [74], regardless of their levels of health literacy.

Lastly, the results with education as a predictor of fruit and vegetable consumption via health literacy also showed inconsistencies with previous studies, as we found a negative direct effect of education on fruit and vegetable consumption, indicating that individuals with higher education have lower levels of fruit and vegetable intake. Previous studies emphasized that the highly educated have higher levels of fruit and vegetable consumption (e.g., [66,75,76]). However, the indirect effect of education on fruit and vegetable consumption via health literacy was positive and significant. On the other hand, the total effects model was not significant. Therefore, we can only partly confirm the results from previous studies, as our findings show that individuals with higher education have higher levels of fruit and vegetable consumption only when mediated by higher health literacy levels (e.g., [3,67]). This suggests that education may enhance healthy lifestyle practices, human capital, and dietary knowledge, while awareness of nutrition’s role in health is generally lower among individuals with less education and lower economic status.

Our study also has several limitations. Firstly, it is a cross-sectional study, which does not permit us to establish causality in our findings. Secondly, our study relies on self-reported measures for dietary intake and health literacy, which may introduce biases, such as overreporting healthy behaviors or misunderstanding survey questions. Additionally, fruit and vegetable intake was measured in terms of the number of days per week they were consumed, without specifying quantities or separating fruits from vegetables. While this approach provided us with an indicator of dietary behavior [77,78,79], it does not account for variations in portion sizes or types of produce consumed, which may influence dietary quality. Thirdly, the coefficients of determination (R^2^) in our mediation analyses were low, indicating that additional factors are likely linked to levels of fruit and vegetable consumption, with health literacy being only one of them. Finally, using broad categories for age, education, and financial stability may oversimplify the nuanced experiences within these groups, potentially masking critical subgroup differences.

In terms of future research, our study has specific cultural and socioeconomic contexts, as data were gathered in Slovenia. Because of this, the results may not be generalizable to other regions or populations. Future studies should therefore examine the mediating role of health literacy in the relationship between demographic and socioeconomic differences in other parts of the world. Furthermore, they should examine age and education as predictors more closely, given the inconsistent effects found in our study. For example, they should investigate possible reasons why older individuals consume more fruits and vegetables despite having lower health literacy levels. Studies should also include additional variables, such as physical activity and accessibility to fresh produce, to provide a more comprehensive understanding of dietary behavior with health literacy as a mediating factor.

The main implication of our study is that nutrition education should target broader segments of society, including marginalized groups such as older adults, individuals with lower education levels, and those with lower incomes. These groups often lack awareness of the importance of nutrition in improving health, which increases their morbidity and mortality. The results also emphasize the need to prioritize health literacy in public health interventions. For example, educational programs could promote fruit and vegetable intake across different demographic groups, with a particular focus on younger populations. There is also a need for policies that improve access to affordable, high-quality products such as fruits and vegetables for financially unstable populations. Therefore, policymakers, educators, and healthcare workers should adapt their efforts and communication strategies based on recent research to improve health literacy in the general population.

## 5. Conclusions

This study highlights the role of health literacy, which mediates the relationship between socioeconomic and demographic factors and fruit and vegetable consumption. Health literacy predicts higher levels of fruit and vegetable consumption. Furthermore, being female and having financial stability also predict higher levels of fruit and vegetable consumption, both with and without the influence of health literacy. These groups also show higher levels of health literacy. Older people also consume higher levels of fruits and vegetables despite lower levels of health literacy. Higher-educated individuals consume more fruits and vegetables only when they also have high levels of health literacy. Policymakers should address health literacy disparities through targeted public health strategies, particularly for financially disadvantaged, less-educated, and younger individuals.

## Figures and Tables

**Table 1:** Descriptive statistics.

		f	%
Gender	Female	1804	46.3
	Male	1556	53.7
Education	No formal education	7	0.2
	Incomplete primary education	49	1.5
	Primary education	431	12.8
	Lower or secondary vocational education	599	17.8
	Secondary technical education	851	25.3
	Gymnasium	383	11.4
	Higher vocational education	309	9.2
	Higher professional education, BA	223	6.6
	Higher university education, MA	284	11.4
	Specialization	30	0.9
	Master of Science or PhD	94	2.8
Ability to pay bills	Very easy	213	6.4
	Easy	1750	52.5
	Difficult	1159	34.8
	Very difficult	212	6.4
Fruit/vegetable intake	Not at all/Never	21	0.63
	Less than 1 day	28	0.83
	1 day	68	2.02
	2 days	90	2.68
	3 days	194	5.77
	4 days	204	6.07
	5 days	322	9.58
	6 days	308	9.17
	7 days	2106	62.72
		M	SD
	Age	51.64	17.69
	Fruit/vegetable intake	6.93	1.74
	Health literacy	68.17	14.34

Notes: M = mean; SD = standard deviation; f = frequency.

**Table 2:** Descriptive statistics and correlations between variables.

	M	SD	1	2	3	4	5
1. Gender (female)	1.54	0.49	-	-	-	-	-
2. Age	51.64	17.69	0.08 **	-	-	-	-
3. Education	5.86	2.13	0.05 *	−0.33 **	-	-	-
4. Economic difficulties	2.41	0.71	0.05 **	0.07 **	−0.34 **	-	-
5. Health literacy	68.17	14.34	0.04 **	−0.23 **	0.29 **	−0.27 **	-
6. Fruit/vegetable intake	9.93	1.74	0.14 **	0.19 **	−0.05 **	−0.08 **	0.08 **

Note: * *p* < 0.05; ** *p* < 0.01. All correlations are Spearman’s rho coefficients (ρ), except for correlations involving gender, which are point-biserial correlations (r_pb_).

**Table 3:** The mediating role of health literacy in the relationship between predictors and fruit and vegetable intake.

Predictor	Predictor → Health Literacy (a; β)	Health Literacy → FV Intake (b; β)	Direct Effect (c’; β)	Indirect Effect (a × b; β)	Total Effect (c’ + a × b; β)	R^2^
Gender	1.137 * [0.176, 2.098]	0.011 ** [0.007, 0.015]	0.480 ** [0.362, 0.598]	0.012 * [0.001, 0.024]	0.492 ** [0.374, 0.611]	0.027
Age	−0.206 ** [−0.233, −0.179]	0.018 ** [0.013, 0.022]	0.019 ** [0.016, 0.022]	−0.004 ** [−0.005, −0.003]	0.015 ** [0.012, 0.018]	0.043
Education	1.929 ** [1.689, 2.169]	0.013 ** [0.008, 0.017]	−0.030 * [−0.058, −0.001]	0.025 ** [0.016, 0.034]	−0.005 [−0.033, 0.023]	0.010
Ability to Pay Bills	−5.740 ** [−6.473, −5.007]	0.009 ** [0.004, 0.013]	−0.166 ** [−0.256, −0.076]	−0.049 ** [−0.075, −0.023]	−0.215 ** [−0.303, −0.127]	0.012

Notes: * *p* < 0.05; ** *p* < 0.01; 95% percent confidence intervals (CI) are presented in brackets; FV intake = fruit and vegetable intake; β = unstandardized regression coefficient; R^2^ = proportion of variance explained.

## Data Availability

The datasets presented in this article are not readily available due to legal project requirements established by NIJZ. Requests to access the datasets should be directed to the National Institute of Public Health Slovenia at: https://nijz.si/.

## Supplementary Materials

The following supporting information can be downloaded at https://www.mdpi.com/article/10.3390/foods14030378/s1, Table S1: The mediating role of health literacy in the relationship between predictors and fruit and vegetable intake (single model). Table S2: Spearman’s (ρ) correlations between variables among men and women.
