# Supplementary material for: Does Health Literacy Mediate Sociodemographic and Economic Inequalities in Fruit and Vegetable Intake? An Analysis of Slovenian National HLS19 Survey Data
Source: Foods. 2025 Jan 24;14(3):378. doi: 10.3390/foods14030378 (PMC11817291; doi:10.3390/foods14030378)
Supplement: Supplementary file 1 [file foods-14-00378-s001.zip › foods-3386149-supplementary.pdf]

Supplementary materials

Table S1. The mediating role of health literacy in the relationship between predictors and fruit and vegetable intake (single-model)

Predictor	Predictor → Health Literacy (a)	Health Literacy → FV Intake (b)	Direct Effect (c')	Indirect Effect (a × b)	Total Effect (c' + a × b)
**Gender**	1.757** [0.859, 2.655]	0.014** [0.010, 0.019]	0.434** [0.316, 0.551]	0.025** [0.010, 0.039]	0.458** [0.341, 0.575]
**Age**	-0.166** [-0.193, -0.140]	0.014** [0.010, 0.019]	0.018** [0.014, 0.021]	-0.002** [-0.003, -0.002]	0.015** [0.012, 0.019]
**Education**	0.922** [0.678, 1.167]	0.014** [0.010, 0.019]	-0.016 [-0.045, 0.014]	0.013** [0.008, 0.018]	-0.003 [-0.032, 0.027]
**Ability to Pay Bills**	-4.615** [-5.343, -3.888]	0.014** [0.010, 0.019]	-0.193** [-0.284, -0.103]	-0.065** [-0.088, -0.042]	-0.258** [-0.347, -0.169]

Notes: * $p < 0.05$; ** $p < 0.01$.; 95% percent confidence intervals (CI) are presented in brackets; FV intake = fruit and vegetable intake; $R^2 = 0.063$.

Table S2. Spearman's (ρ) correlations between variables among men and women

		1	2	3	4
**Men**					
	1. Age	-	-	-	-
	2. Education	-0.24**	-	-	-
	3. Economic difficulties	0.04	-0.33**	-	-
	4. Health literacy	-0.19**	0.22**	-0.24**	-
	5. Fruit/vegetable intake	0.18**	-0.09**	-0.08**	0.08**
**Women**					
	1. Age	-	-	-	-
	2. Education	-0.43**	-	-	-
	3. Economic difficulties	0.10**	-0.37**	-	-
	4. Health literacy	-0.29**	0.35**	-0.30**	-
	5. Fruit/vegetable intake	0.19**	-0.03	-0.09**	0.06*

Notes: * $p < 0.05$; ** $p < 0.01$.
